# Supplementary figures and images for: Decrease in the prevalence of antimicrobial resistance in Escherichia coli isolates of Canadian turkey flocks driven by the implementation of an antimicrobial stewardship program
Source: PLoS One. 2023 Jul 24;18(7):e0282897. doi: 10.1371/journal.pone.0282897 (PMC10365295; doi:10.1371/journal.pone.0282897)

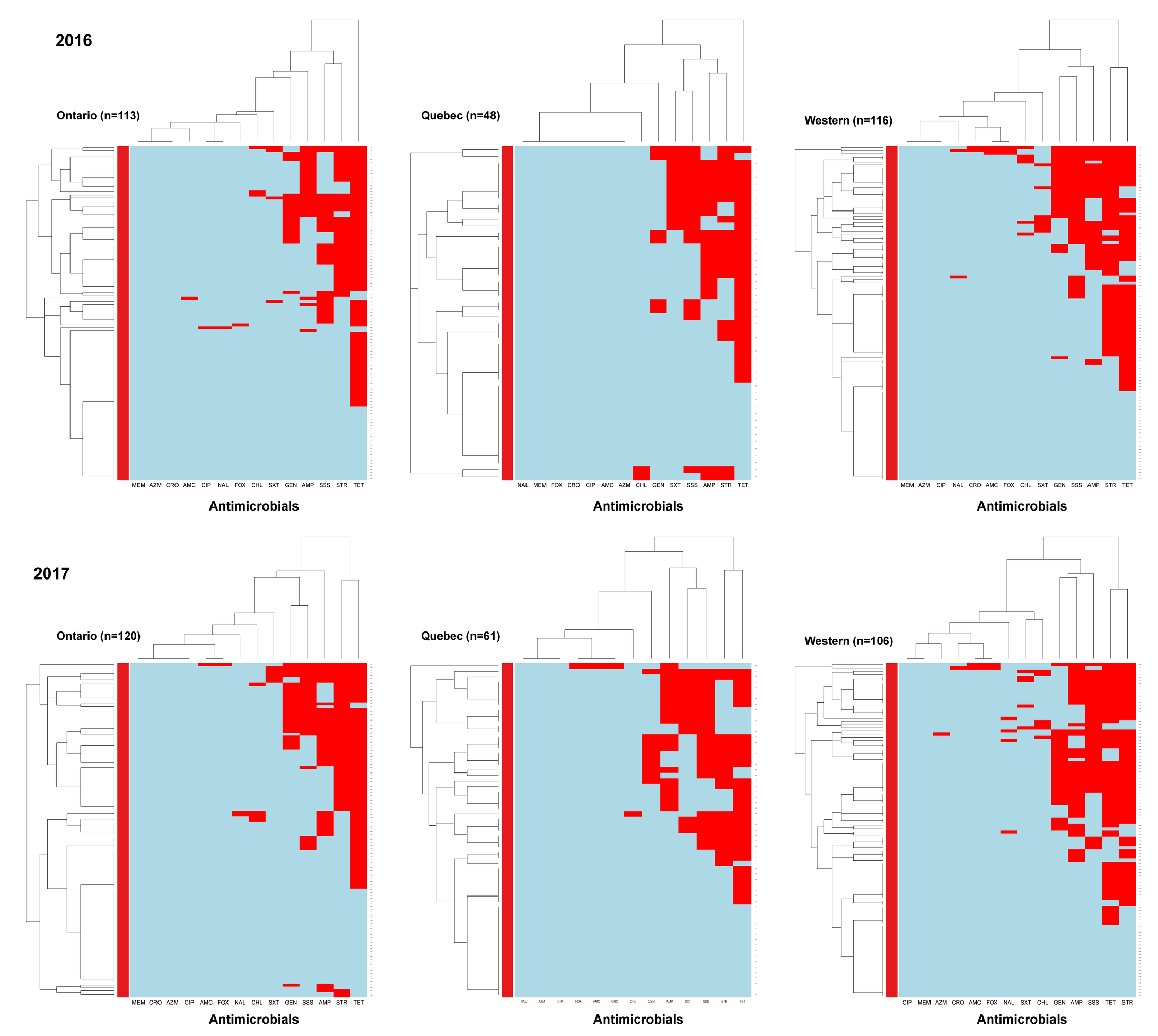

Supplement: S1 Fig — X-axes represent the antimicrobial classes: amoxicillin (AMC), ampicillin (AMP), azithromycin (AZM), chloramphenicol (CHL), ciprofloxacin (CIP), ceftriaxone (CRO), cefoxitin (FOX), gentamicin (GEN), meropenem (MEM), nalidixic acid (NAL), Sulfisoxazole (SSS), Streptomycin (STR), Trimethoprim-Sulfamethoxazole (SXT), Tetracyclines (TET). Y-axes represent the E. coli isolates included in this study. The blue color depicts susceptibility, and the red color illustrates resistant patterns. (TIF) [file pone.0282897.s001.tif]

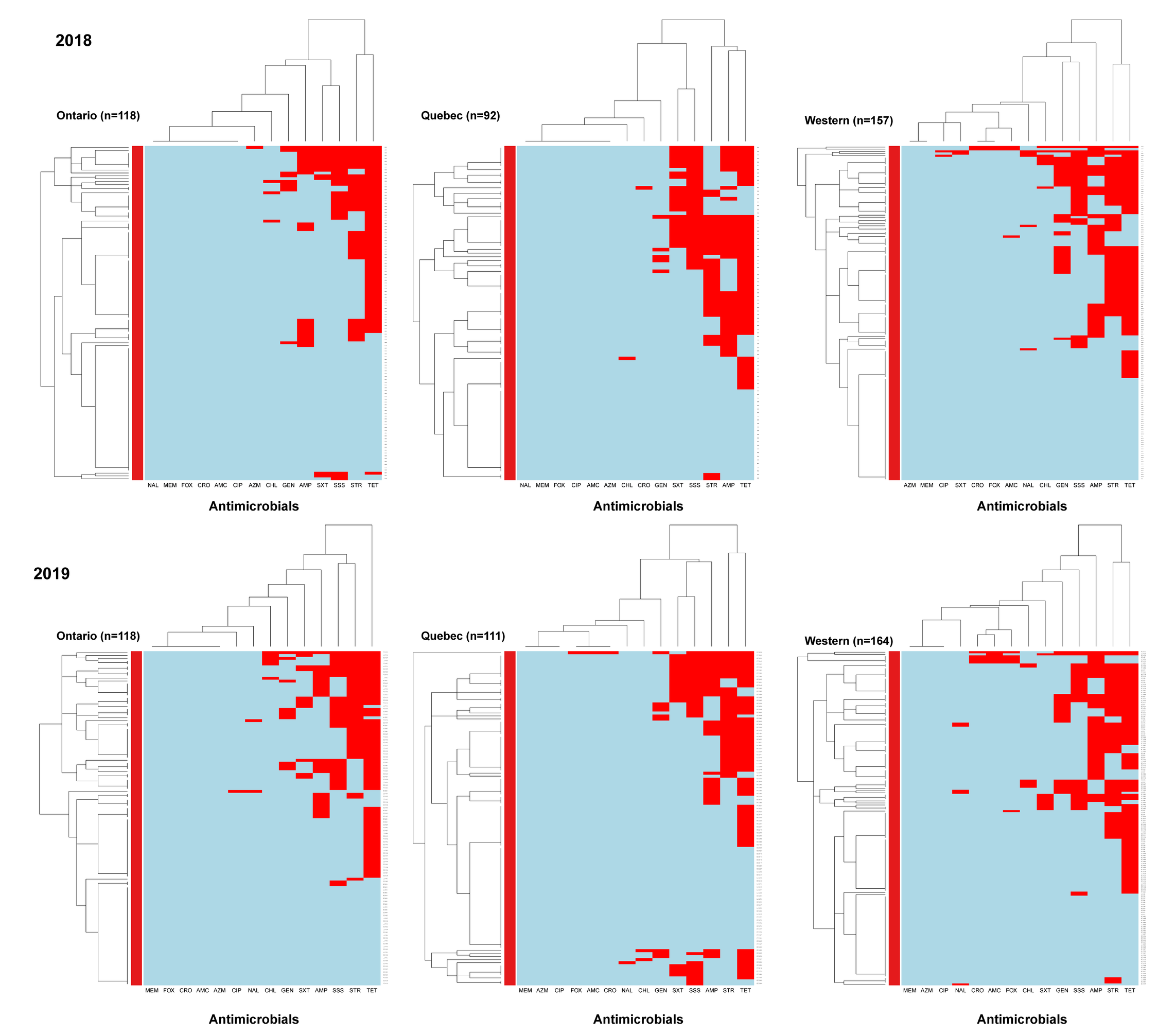

Supplement: S2 Fig — X-axes represent the antimicrobial classes: amoxicillin (AMC), ampicillin (AMP), azithromycin (AZM), chloramphenicol (CHL), ciprofloxacin (CIP), ceftriaxone (CRO), cefoxitin (FOX), gentamicin (GEN), meropenem (MEM), nalidixic acid (NAL), Sulfisoxazole (SSS), Streptomycin (STR), Trimethoprim-Sulfamethoxazole (SXT), Tetracyclines (TET). Y-axes represent the E. coli isolates included in this study. The blue color depicts susceptibility, and the red color illustrates resistant patterns. (TIF) [file pone.0282897.s002.tif]

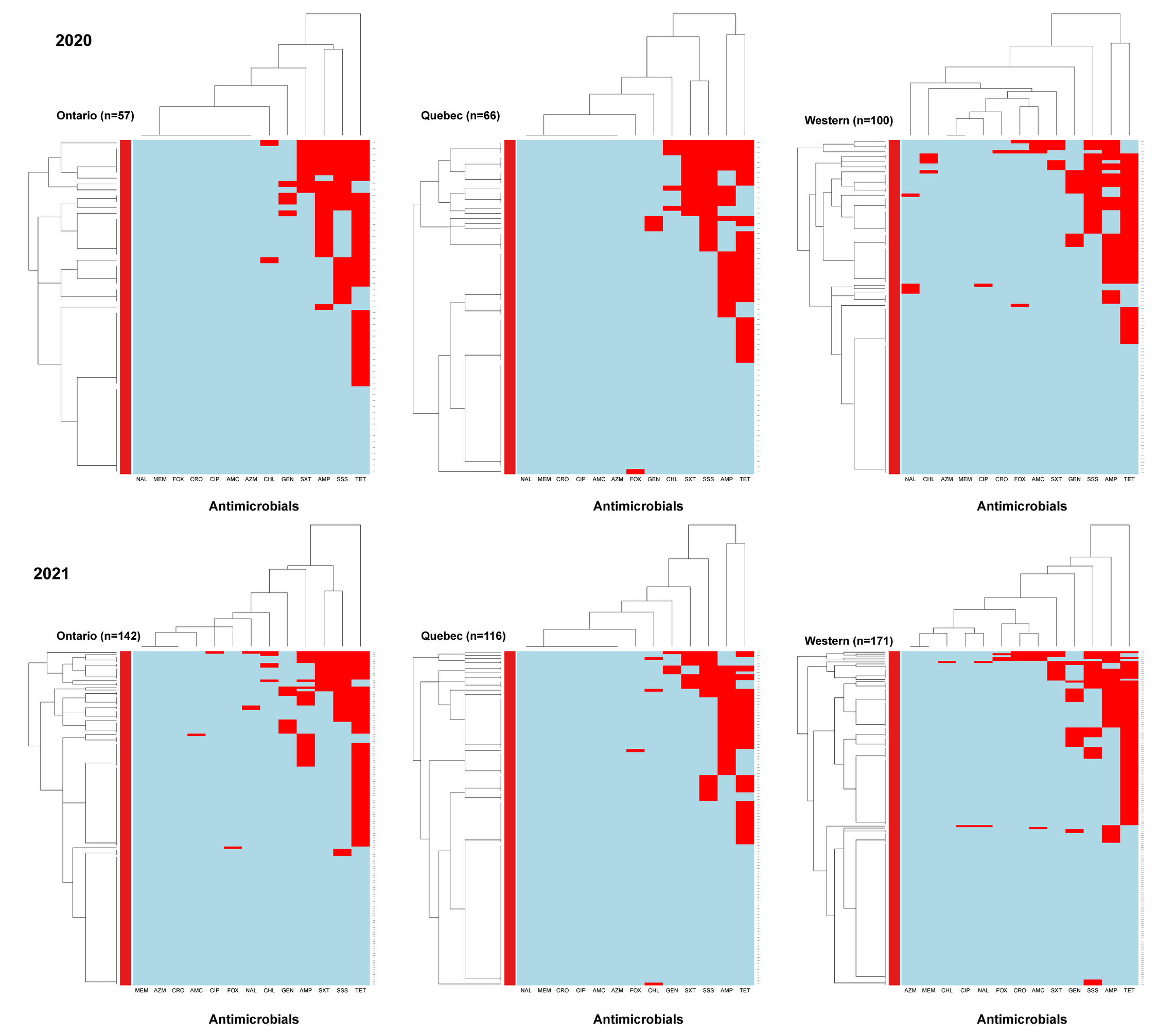

Supplement: S3 Fig — X-axes represent the antimicrobial classes: amoxicillin (AMC), ampicillin (AMP), azithromycin (AZM), chloramphenicol (CHL), ciprofloxacin (CIP), ceftriaxone (CRO), cefoxitin (FOX), gentamicin (GEN), meropenem (MEM), nalidixic acid (NAL), Sulfisoxazole (SSS), Streptomycin (STR), Trimethoprim-Sulfamethoxazole (SXT), Tetracyclines (TET). Y-axes represent the E. coli isolates included in this study. The blue color depicts susceptibility, and the red color illustrates resistant patterns. (TIF) [file pone.0282897.s003.tif]

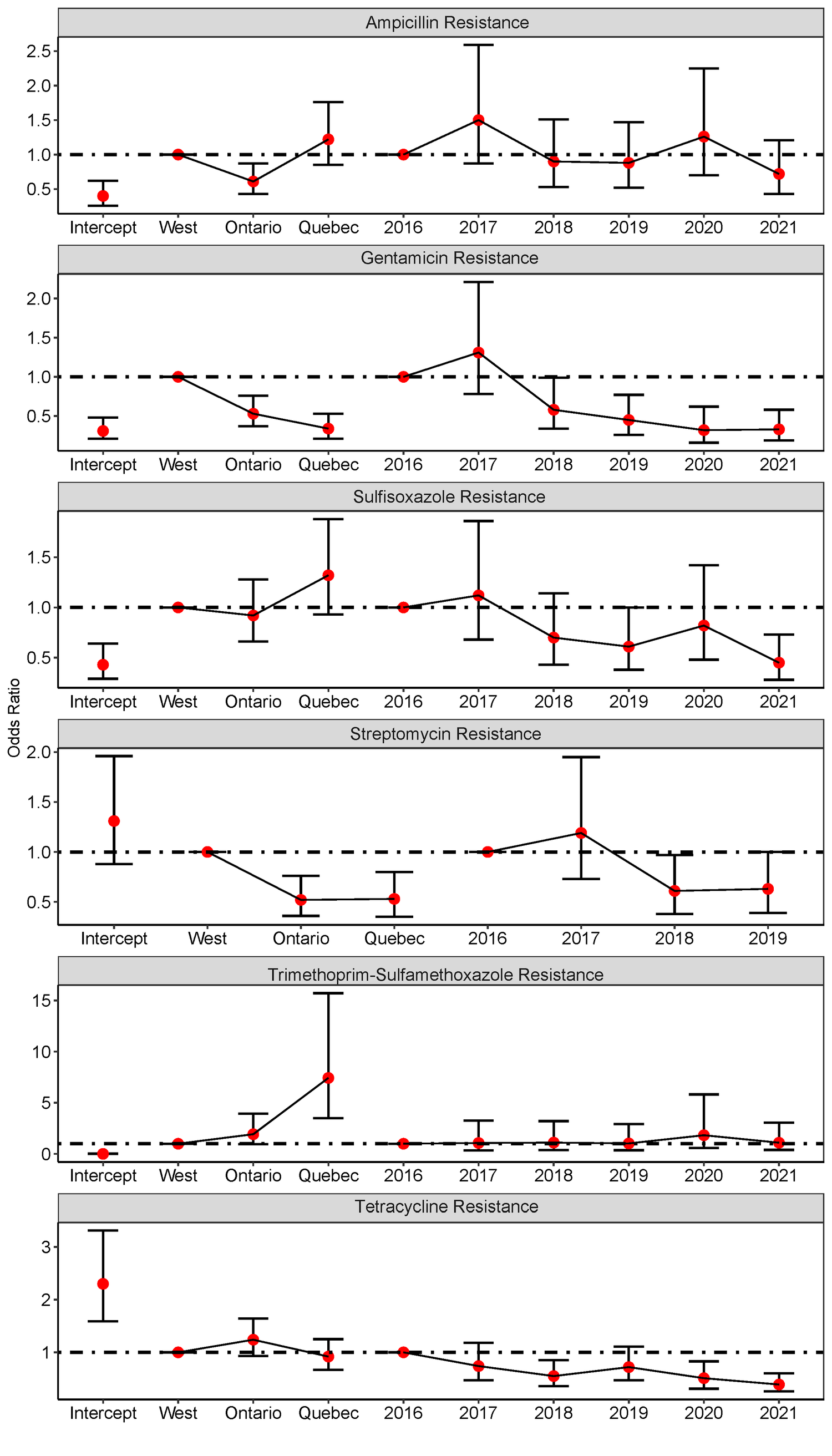

Supplement: S4 Fig — (TIF) [file pone.0282897.s004.tif]
