# Supplementary material for: Decrease in the prevalence of antimicrobial resistance in Escherichia coli isolates of Canadian turkey flocks driven by the implementation of an antimicrobial stewardship program
Source: PLoS One. 2023 Jul 24;18(7):e0282897. doi: 10.1371/journal.pone.0282897 (PMC10365295; doi:10.1371/journal.pone.0282897)
Supplement: S1 Table — (DOCX) [file pone.0282897.s005.docx]

Table S1: Antimicrobial use numerator and denominator input parameters and the number of turkey flocks enrolled in this study from 2016 to 2021.

| Parameters | Sampling Year | | | | | |
| --- | --- | --- | --- | --- | --- | --- |
|  | **2016** | **2017** | **2018** | **2019** | **2020** | **2021** |
| Number of turkey flocks | 72 | 74 | 95 | 98 | 61 | 110 |
| Numerator parameters |  |  |  |  |  |  |
| Total mg | 3,021 | 3,141 | 3,269 | 4,827 | 1,446 | 1,898 |
| Mean flock-level mg | 42 | 42 | 34 | 49 | 24 | 17 |
| Total turkey population | 558,396 | 550,587 | 608,994 | 687,360 | 390,487 | 677,018 |
| Denominator parameters |  |  |  |  |  |  |
| Mean flock-level population | 7,755 | 7,440 | 6,410 | 7,014 | 6,401 | 6,155 |
| Mean flock-level bodyweights (Kg) | 10 | 10 | 10 | 10 | 10 | 11 |
| Total Kg animal biomass | 5,011,422 | 5,070,026 | 5,582,249 | 6,353,551 | 3,717,665 | 6,914,167 |
| Mean flock-level animal biomass | 69,603 | 68,514 | 58,761 | 64,832 | 60,945 | 62,856 |
