# Supplementary material for: Decrease in the prevalence of antimicrobial resistance in Escherichia coli isolates of Canadian turkey flocks driven by the implementation of an antimicrobial stewardship program
Source: PLoS One. 2023 Jul 24;18(7):e0282897. doi: 10.1371/journal.pone.0282897 (PMC10365295; doi:10.1371/journal.pone.0282897)
Supplement: S2 Table — (DOCX) [file pone.0282897.s006.docx]

S2 Table. Summary of the quantity of AMU per year by region.

| **Region** | **Year** | | Quantity of antimicrobial use (mg/kg biomass) | | | | | |
| --- | --- | --- | --- | --- | --- | --- | --- | --- |
|  |  |  | **Total** | | **Mean (SD)** | | **Range** | |
| Ontario | 2016 | | 1414 | | 47.13 (44.31) | | (0-166) | |
|  | 2017 | | 1531 | | 46.39 (50.79) | | (0-181) | |
|  | 2018 | | 549 | | 30.5 (52.41) | | (0-201) | |
|  | 2019 | | 988 | | 52 (76.21) | | (0-322) | |
|  | 2020 | | 464 | | 27.29 (29.81) | | (0-102) | |
|  | 2021 | | 334 | | 9.28 (14.73) | | (0-57) | |
| Quebec | 2016 | | 401 | | 33.42 (24.38) | | (0-72) | |
|  | 2017 | | 365 | | 26.07 (34.04) | | (0-109) | |
|  | 2018 | | 1359 | | 36.73 (45.81) | | (0-153) | |
|  | 2019 | | 1612 | | 42.42 (53.89) | | (0-161) | |
|  | 2020 | | 607 | | 33.72 (39.50) | | (0-112) | |
|  | 2021 | | 148 | | 5.10 (18.04) | | (0-79) | |
| Western | 2016 | | 1213 | | 40.43 (26.96) | | (0-114) | |
|  | 2017 | | 1274 | | 47.18 (36.91) | | (0-171) | |
|  | 2018 | | 1373 | | 34.33 (30.59) | | (0-111) | |
|  | 2019 | | 2235 | | 54.51 (43.62) | | (0-243) | |
|  | 2020 | | 423 | | 16.27 (25.62) | | (0-82) | |
|  | 2021 | | 1452 | | 32.27 (50.54) | | (0-228) | |
| Grand Total | | 17742 | | 34.79 (43.19) | | (0-322) | |  |
