# Supplementary material for: Decrease in the prevalence of antimicrobial resistance in Escherichia coli isolates of Canadian turkey flocks driven by the implementation of an antimicrobial stewardship program
Source: PLoS One. 2023 Jul 24;18(7):e0282897. doi: 10.1371/journal.pone.0282897 (PMC10365295; doi:10.1371/journal.pone.0282897)
Supplement: S3 Table — (DOCX) [file pone.0282897.s007.docx]

S3 Table. Summary of the AMR in *E. coli* isolates of Canadian turkey flocks per year by region.

| **Region** | **Year** | Resistance to Antimicrobials^a^ | | | | | | | | | | | | | | MDR^b^ |
| --- | --- | --- | --- | --- | --- | --- | --- | --- | --- | --- | --- | --- | --- | --- | --- | --- |
|  |  | **AMC** | **AMP** | **AZM** | **CHL** | **CIP** | **CRO** | **FOX** | **GEN** | **MEM** | **NAL** | **SSS** | **ST** | **SXT** | **TET** |  |
| Ontario | 2016 | 1 | 27 | 0 | 3 | 1 | 0 | 1 | 21 | 0 | 1 | 33 | 45 | 4 | 83 | 41 |
|  | 2017 | 1 | 41 | 0 | 5 | 0 | 0 | 1 | 29 | 0 | 2 | 32 | 56 | 6 | 79 | 42 |
|  | 2018 | 0 | 23 | 1 | 3 | 0 | 0 | 0 | 9 | 0 | 0 | 27 | 39 | 13 | 67 | 29 |
|  | 2019 | 0 | 26 | 0 | 6 | 1 | 0 | 0 | 10 | 0 | 2 | 33 | 41 | 11 | 72 | 34 |
|  | 2020 | 0 | 20 | 0 | 2 | 0 | 0 | 0 | 4 | 0 | 0 | 20 | - | 9 | 36 | 14 |
|  | 2021 | 1 | 27 | 0 | 5 | 1 | 0 | 1 | 10 | 0 | 3 | 33 | - | 17 | 76 | 24 |
| Quebec | 2016 | 0 | 20 | 0 | 2 | 0 | 0 | 0 | 6 | 0 | 0 | 18 | 21 | 12 | 33 | 23 |
|  | 2017 | 1 | 21 | 0 | 1 | 0 | 1 | 1 | 10 | 0 | 0 | 27 | 22 | 15 | 35 | 28 |
|  | 2018 | 0 | 39 | 0 | 1 | 0 | 1 | 0 | 5 | 0 | 0 | 34 | 34 | 24 | 53 | 36 |
|  | 2019 | 1 | 33 | 0 | 2 | 0 | 1 | 1 | 9 | 0 | 1 | 31 | 39 | 20 | 66 | 36 |
|  | 2020 | 0 | 24 | 0 | 5 | 0 | 0 | 1 | 3 | 0 | 0 | 22 | - | 15 | 34 | 16 |
|  | 2021 | 0 | 38 | 0 | 3 | 0 | 0 | 1 | 4 | 0 | 0 | 25 | - | 10 | 48 | 14 |
| Western | 2016 | 3 | 36 | 0 | 6 | 0 | 2 | 3 | 29 | 0 | 2 | 42 | 67 | 8 | 74 | 46 |
|  | 2017 | 2 | 45 | 1 | 6 | 0 | 1 | 2 | 29 | 0 | 4 | 44 | 68 | 5 | 66 | 53 |
|  | 2018 | 3 | 46 | 0 | 8 | 2 | 2 | 2 | 36 | 0 | 5 | 41 | 67 | 2 | 86 | 52 |
|  | 2019 | 6 | 55 | 0 | 6 | 0 | 5 | 6 | 23 | 0 | 5 | 38 | 72 | 9 | 103 | 61 |
|  | 2020 | 4 | 35 | 0 | 4 | 1 | 1 | 3 | 11 | 0 | 4 | 27 | - | 6 | 50 | 25 |
|  | 2021 | 6 | 45 | 0 | 1 | 1 | 5 | 3 | 22 | 0 | 2 | 32 | - | 13 | 85 | 32 |
| Grand Total | | **29** | **601** | **2** | **69** | **7** | **19** | **26** | **270** | **0** | **31** | **559** | **571** | **199** | **1146** | **606** |
| ^a^Resistance to amoxicillin (AMC), ampicillin (AMP), azithromycin (AZM), chloramphenicol (CHL), ciprofloxacin (CIP), ceftriaxone (CRO), cefoxitin (FOX), gentamicin (GEN), meropenem (MEM), nalidixic acid (NAL), Sulfisoxazole (SSS), Streptomycin (STR), Trimethoprim-Sulfamethoxazole (SXT), Tetracyclines (TET).  ^b^ MDR: Multidrug resistance (≥ 3 antimicrobial classes) | | | | | | | | | | | | | | | | |
